# Length of Lecture Terms in Different Colleges

**Published:** 1848-10

**Authors:** 


*Length of Lecture Terms in different Colleges.*—We copy the following from the Medical Examiner, with a few additions to the list made by us:—

| Institution. | No. of Professors. | Length of Term. |
|---|---|---|
| Harvard, Boston, | 7 | Four months. |
| Berkshire, Mass. | 6 | Fourteen weeks. |
| Dartmouth, N. H. | 6 | Fourteen weeks. |
| Castleton, Vermont, | 6 | Four months. |
| Yale, Conn. | 6 | Sixteen weeks. |
| University of New-York, | 6 | Four months. |
| Buffalo, " | 7 | Twenty weeks. |
| Geneva, " | 6 | Sixteen weeks. |
| Albany, " | 7 | Sixteen weeks. |
| Col. Phy. and Surg." | 7 | Five months and a half. |
| University of Penn. | 7 | Five months and a half. |
| Jefferson Med. Col. | 7 | Five months and a half. |
| University of Maryland, | 6 | Five months and a half. |
| Hampden Sidney, Va. | 6 | Nearly five months. |
| University of Virginia, | 3 | Eight months. |
| Winchester Med. Col. | 5 | Seven months. |
| Med. Col. of S. C. | 7 | Five months. |
| Georgia Med. Col. | 7 | Five months. |
| Memphis Med. Col. | 7 | Four months. |
| Transylvania Un., Ky. | 7 | Four months. |
| Un. of Louisville, " | 7 | Four months and a half. |
| St. Louis. Univ. of Mo. | 8 | Four months and a half. |
| Univ. State of Missouri, | 6 | Four months and a half. |
| Ohio Med. Col. | 6 | Four months. |
| Cleveland Med. College, | 6 | Sixteen weeks. |
| Starling Med. College, | 6 | Sixteen weeks. |

